# New advances in understanding thyroid-associated ophthalmopathy and the potential role for insulin-like growth factor-I receptor

**DOI:** 10.12688/f1000research.12787.1

**Published:** 2018-02-01

**Authors:** Terry J Smith

**Affiliations:** 1 Department of Ophthalmology and Visual Sciences, Kellogg Eye Center and Division of Metabolism, Endocrinology and Diabetes, Department of Internal Medicine, University of Michigan Medical School, Ann Arbor, MI 48105, USA

**Keywords:** opthamology, thyroid, Insulin-like growth factor

## Abstract

Thyroid-associated ophthalmopathy (TAO), a localized periocular manifestation of the autoimmune syndrome known as Graves’ disease, remains incompletely understood. Discussions of its pathogenesis are generally focused on the thyrotropin receptor, the proposed role for which is supported by substantial evidence. Considerations of any involvement of the insulin-like growth factor-I receptor (IGF-IR) in the disease are frequently contentious. In this brief, topically focused review, I have attempted to provide a balanced perspective based entirely on experimental results that either favor or refute involvement of IGF-IR in TAO. Discussion in this matter seems particularly timely since the currently available treatments of this disfiguring and potentially sight-threatening disease remain inadequate. Importantly, no medical therapy has thus far received approval from the US Food and Drug Administration. Results from a very recently published clinical trial assessing the safety and efficacy of teprotumumab, an inhibitory human anti–IGF-IR monoclonal antibody, in active, moderate to severe TAO are extremely encouraging. That double-masked, placebo-controlled study involved 88 patients and revealed unprecedented clinical responses in the improvement of proptosis and clinical activity as well as a favorable safety profile. Should those results prove reproducible in an ongoing phase III trial, therapeutic inhibition of IGF-IR could become the basis for paradigm-shifting treatment of this vexing disease.

## Introduction

**Figure 1.**
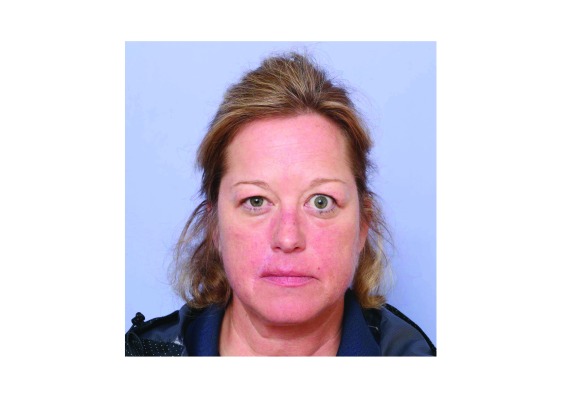
Facial appearance of a patient with thyroid-associated ophthalmopathy. In this case, involvement of the tissues surrounding the eye is dramatically asymmetrical. Note the upper eyelid edema, eyelid retraction and the facial redness.

This brief review is intended to clarify the current understanding of thyroid-associated ophthalmopathy (TAO), its pathogenesis, and how identification of fundamental disease mechanisms might ultimately dictate its treatment. TAO is characterized by inflammation and remodeling of the soft tissues of the orbit and upper face ( Figure 1) ^1^. It is a manifestation of Graves’ disease (GD), a systemic autoimmune syndrome ^2^. About 40% of patients with GD develop clinically significant TAO ^1^. Many of those manifesting ocular disease require local supportive therapy. Others with more severe TAO require aggressive therapies such as systemic glucocorticoids and external beam orbital radiation ^3, 4^. These options come with substantial side effects that limit their dosage and treatment duration. Outcomes of remedial surgeries, usually reserved for patients with the most severe disease, are unpredictable and can reactivate the inflammatory, progressive (active) phase of TAO ^5^. In GD, the thyroid becomes overactive and produces excessive thyroid hormones, a consequence of the generation of activating antibodies (thyroid-stimulating immunoglobulin, or TSI) targeting the thyrotropin receptor (thyroid-stimulating hormone receptor, or TSHR) ^6^. The immunological events underlying disease manifestations occurring in the thyroid and orbit in GD are presumed to be closely related if not identical. It is possible that the orbit and thyroid share the expression of an autoantigen (or autoantigens) and that immunoreactivity within the thyroid initiates events occurring in the orbit. This attractive theory has yet to be proven, and many details of disease pathogenesis occurring in both tissues remain uncertain. While hyperthyroidism is treated effectively with decades-old approaches, clinical management of TAO remains largely unsatisfactory and therefore represents an important unmet public health need. None of the existing medical therapies for TAO has achieved registration by the US Food and Drug Administration. Thus, it will be necessary to identify better therapeutic targets for the orbital disease if we are to advance the care of patients with TAO.

## Thyroglobulin as the initially identified orbital autoantigen candidate in thyroid-associated ophthalmopathy

**Figure 2.**
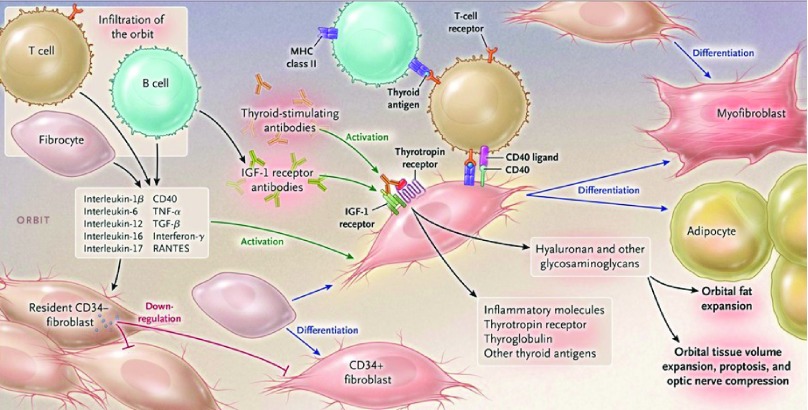
Pathogenesis of Graves’ disease affecting the orbit. The orbit becomes infiltrated by B and T cells and CD34 ^+^ fibrocytes uniquely in thyroid-associated ophthalmopathy. Bone marrow-derived fibrocytes express several proteins traditionally considered “thyroid-specific”. They can differentiate into CD34 ^+^ fibroblasts which can further develop into myofibroblasts or adipocytes depending upon the molecular cues they receive from the tissue microenvironment. CD34 ^+^ fibroblasts cohabit the orbit with residential CD34 ^−^ fibroblasts. These heterogeneous populations of orbital fibroblasts can produce cytokines under basal and activated states. These include interleukin-1beta (IL-1β), IL-6, IL-8, IL-10, and IL-16; IL-1 receptor antagonists; tumor necrosis factor-alpha; the chemokine known as “regulated on activation, normal T expressed and secreted” (or RANTES); CD40 ligand; and several other cytokines and chemokines. These cytokines can act on infiltrating and residential cells. Like fibrocytes, CD34 ^+^ fibroblasts express thyrotropin receptor, thyroglobulin, and other thyroid proteins but at substantially lower levels. Thyroid-stimulating immunoglobulins and potentially other autoantibodies directed specifically at the insulin-like growth factor-I receptor activate the thyrotropin/insulin-like growth factor receptor-I complex, resulting in the activation of several downstream signaling pathways and expression of target genes. Orbital fibroblasts synthesize hyaluronan, leading to increased orbital tissue volume. This expanded tissue can result in proptosis and optic nerve compression. Orbital fat also expands from *de novo* adipogenesis. IGF-I, insulin-like growth factor I; MHC, major histocompatibility complex; RANTES, regulated on activation, normal T cell expressed and secreted; TGF-β, transforming growth factor-beta; TNF-α, tumor necrosis factor-alpha. Reprinted with permission from the Massachusetts Medical Society ^2^.

Current understanding of TAO involves a complex set of interactions between the professional immune system and the residential tissues in the orbit ( Figure 2). A long-held view of the underpinning connection between GD occurring within the thyroid and orbit focuses on the potential for a shared autoantigen. Thyroglobulin (Tg) was the earliest candidate antigen emerging from the work of Kriss ^7^. His findings suggested that Tg could accumulate in the TAO orbit. He proposed that the protein was transported in GD through the lymphatic system from the thyroid to the orbit. He apparently never considered the potential for localized, ectopic Tg synthesis in orbital connective tissues. His theoretical model for TAO pathogenesis remained unembellished for several decades. Another laboratory group reported that they could not detect Tg in extraocular muscle ^8^. Much more recently, a group in Italy ^9, 10^ demonstrated the Tg-binding capacity of orbital fibroblasts. That same group detected Tg in orbital fibroadipose tissue from the majority of patients (four out of six) with TAO whom they studied. Furthermore, they could detect thyroxine residues in most of the samples and interpreted that finding as strongly suggestive that orbital Tg was of thyroid origin.

These concepts concerning how Tg might accumulate in the TAO orbit have been modified substantially following the discovery by Douglas *et al*. of fibrocytes in the TAO orbit ^11^. These investigators reported increased abundance of circulating CD34 ^+^ fibrocytes in GD. Fibrocytes derive from the monocyte lineage and appear to play important roles in tissue remodeling and wound healing ^12^. They have been implicated in TAO where they infiltrate connective tissues of the orbit while retaining their CD34 ^+^CXCR4 ^+^Collagen I ^+^ phenotype. They cohabitate the fibroblast population in the fatty tissues with residential CD34 ^−^ fibroblasts. Fibrocytes have also been associated with several other autoimmune diseases, including rheumatoid arthritis, scleroderma, diabetes mellitus, and inflammatory bowel disease ^13– 15^. Fibrocytes can differentiate into myofibroblasts, adipocytes, osteoblasts, and chondrocytes ^16^. With regard to TAO, the cells appear to account, at least in part, for the cellular heterogeneity found in the orbit ^11^. Fibrocytes express several proteins heretofore considered to be “thyroid-specific” ^17^. Among these, Tg was found to be synthesized *de novo* by these cells ^18^. The molecule appears to be functional in that it can incorporate iodine. These findings thus have provided a plausible explanation for extra-thyroidal production of Tg and its accumulation in orbital connective tissue in TAO ^7^. Current evidence in aggregate, however, does not support direct involvement of orbital Tg in the pathogenesis of TAO. While anti-Tg antibodies are commonly detected in patients with autoimmune thyroid diseases such as GD, these antibodies appear to be non-pathogenic and are not specific for TAO or GD ^19^. Furthermore, antigen-specific T cells have not been detected infiltrating orbital tissues in the disease. Clearly, further investigation into the consequences of orbital Tg will need to be undertaken before definitive statements can be made about involvement of this protein in ocular GD.

## Thyroid-stimulating hormone receptor as a candidate orbital antigen in thyroid-associated ophthalmopathy

Cloning of the TSHR gene by Parmentier *et al*. ^20^ ushered in a period of active inquiry into its physiological roles and its participation in human disease. The receptor was detected in thyroid epithelium, as anticipated, but also in several adipose tissue depots, albeit at substantially lower levels. These include normal orbital tissue and fat from patients with TAO ^21^. These observations led to the now widely held view that TSHR may represent an important link between the thyroid and orbit in GD. Supporting this concept, TSH and immunoglobulin G from patients with GD (GD-IgG) have been shown to exert actions in cultured orbital fibroblasts and fibrocytes ^11, 22, 23^. The expression of several cytokines can be upregulated as can the accumulation of hyaluronan in orbital fibroblasts ^22– 24^. TSIs can be detected in most patients with TAO, and studies have suggested that the levels of these antibodies correlate with disease severity and activity ^25^. In contrast, a small minority of patients with TAO can present in the euthyroid state with undetectable TSIs ^26^. These unusual cases, some with severe eye disease, may prove powerfully instructive in that they have called into question the notion that TSIs are absolutely required for the development of TAO. They certainly do raise the possibility that another autoantigen and its cognate antibody play a role in the pathogenesis of the disease.

## Thyroid-stimulating hormone receptor immunization in rodent models of Graves’ disease

Efforts to generate animal models of GD date back decades. Some were successful in generating antibody-driven thyroid dysfunction but most lacked orbital pathology. A recently developed mouse created by transferring human TSHR A-subunit to NOD- *H2 ^h4^* mice resulted in euthyroid TSHR-transgenic NOD- *H2 ^h4^* animals spontaneously generating pathogenic anti-human TSHR antibodies and apparently not developing orbital manifestations ^27^. Female mice were more prone to developing these antibodies than were the male animals. Recent progress has been made in the development of more complete animal models of GD based on immunizations with TSHR. One group reported the induction of periocular changes bearing variable resemblance to the pathology commonly observed in human disease ^28^. Their model involved animals immunized with human TSHR A-subunit plasmid delivered intramuscularly followed by electroporation. The phenotypes generated with their experimental protocol are variable and diverge from the typical clinical presentation of human GD and TAO. In their first report, 75% of female BALB/c mice developed hyperthyroidism and detectable TSI ^28^. Thyroids were enlarged and infiltrated with mononuclear cells in a patchy distribution. Orbital tissues exhibited fibrosis and an accumulation of Masson’s trichrome-staining material. A second report ^29^ using what appears to be an identical experiment protocol resulted in profoundly hypothyroid animals with TSHR-blocking antibodies and extraocular muscle infiltrates accompanied by hyaluronan deposition and orbital fat expansion. In the enclosed micrographs of affected orbital tissues, a robust mononuclear cell infiltrate appears to be involving the optic nerve, a feature strikingly uncharacteristic of TAO. A third report involving examination of TSHR-immunized mice from this same group describes parallel studies conducted in two academic centers in different countries using the same protocol and yielding hyperthyroid animals ^30^. Furthermore, the orbital pathology revealed fat expansion, fibrosis, and muscle disruption in the absence of mononuclear cell infiltrates. The authors of these studies conclude that the dominance of stimulatory versus blocking antibodies is random. This point of view appears to lack support of experimental evidence, and the results they have reported thus far suggest that these investigators have simply not yet identified the factors underlying their discrepant results. They rationalize the absence of inflammatory infiltrates in their latest study as consistent with a “hit and run” immune-mediated inflammatory event ^30^. Another research group, also employing intramuscular DNA immunization followed by electroporation, found that a large fraction of their animals developed elevated thyroxine levels, anti-TSHR antibodies, and goiters ^31^. Extraocular muscle and adipose tissue volumes were increased. A different approach, one in which splenocytes from TSHR knockout mice immunized with mouse TSHR A-subunit encoding adenovirus were adoptively transferred to athymic nude mice, resulted in anti-TSHR antibodies and macrophage infiltrates in orbital fat and extraocular muscles ^32^.

In aggregate, these reports describe substantial progress in experimentally inducing phenotypes sharing variable similarity with TAO. They are encouraging; however, concerns have been raised regarding whether rodent models can recapitulate human disease with high fidelity ^33, 34^. Many investigators contend that small rodents may be incapable of adequately approximating human autoimmune disease, the consequence of fundamental differences in immune systems. Small animal preclinical models for TAO could emerge as useful screening tools for therapy development. In any event, well-conceived, properly controlled, and adequately powered therapeutic trials are likely to remain the mainstays for drug candidates as they embark on the road to registration.

## Insulin-like growth factor-I receptor as an active participant in thyroid-associated ophthalmopathy

Important gaps in current understanding of disease pathophysiology and incomplete explanations of the clinical behavior of TAO have prompted consideration of unorthodox mechanisms. For instance, it has not been possible to detect TSI in a small fraction of patients with TAO ^26^, raising questions about the absolute requirement for those antibodies in disease development. Some patients with GD and extremely high levels of TSI develop TAO whereas others with the identical serological profiles do not. Among the candidate molecules currently discussed as potentially involved in the disease is insulin-like growth factor-I receptor (IGF-IR), a widely expressed cell-surface protein found in most tissues and cell types in the human body. Evidence is mounting that IGF-IR not only may play a substantive role in disease development and progression but also may represent an important therapeutic target.

A relationship between the IGF-I pathway and the actions of TSH and TSI was initially demonstrated by Ingbar *et al*. ^35^. They found that IGF-I could enhance the effects of those agents in thyroid cells in culture. This synergy between TSH and IGF-I has been confirmed in several recent reports ^36^. IGF-IR is a membrane-bound tyrosine kinase protein ^37^ that serves as the cognate receptor for both IGF-I and IGF-II. IGF-IR has been implicated in several autoimmune diseases, including type I diabetes mellitus, rheumatoid arthritis, and inflammatory bowel disease ^38^. Its involvement in TAO has been proposed in a two-component theoretical model stemming largely from work performed in our laboratory. First, anti–IGF-IR antibodies have been detected in patients with GD ^39, 40^ and are essentially absent in sera from healthy controls. A major remaining uncertainty is whether those antibodies are active in initiating signaling directly through IGF-IR. Pritchard *et al*. found that GD-IgG could induce genes encoding T-cell chemoattractant cytokines in orbital fibroblasts ^40, 41^. Those effects were mediated through the Akt/FRAP/mTor/p70 ^s6k^ pathways, were rapamycin-sensitive, and could be blocked by inhibitory antibodies targeting IGF-IR and by transfection of orbital fibroblasts with a dominant negative IGF-IR ^40, 41^. In another study, GD-IgG was found to induce the synthesis of hyaluronan in orbital fibroblasts but not in fibroblasts from healthy donors ^24^. Subsequently, TSHR and IGF-IR were found to form a physical and functional signaling complex ^42^. Specifically, signaling downstream from TSHR leading to the activation of Erk is dependent on IGF-IR activity. Thus, the detection of anti–IGF-IR antibodies with apparent receptor-activating effects ^40^ coupled with the interdependence of TSHR/IGF-IR signaling ^42, 43^ provided the necessary rationale for proposing involvement of IGF-IR in TAO. This concept led directly to organizing a therapeutic trial of an inhibitory anti–IGF-IR monoclonal antibody in the treatment of active, moderate to severe TAO ^44^.

Subsequent reports came to a variety of conclusions regarding whether IGF-IR might play a role in TAO. Some have confirmed the dependence of TSHR-initiated signaling on IGF-IR activity by using several different methodologies ^45^. Others have come to different conclusions concerning whether TSHR and IGF-IR act as integrated molecular partners. Moreover, some authors have dismissed the findings of anti–IGF-IR antibodies in GD and TAO ^45, 46^. Their conclusions are based largely on studies using a variety of different experimental models and approaches. Issues such as assay sensitivities, incomplete assessment of treatment duration, and potentially confounding variations in the cellular targets and experimental conditions used, including culture medium content could have led to results and their interpretations differing from those of the initial studies. In general, these authors attribute all of the actions of GD-IgG to the effects of TSIs acting directly on TSHR. No one comments on the parallel findings of Pritchard *et al*. ^47^ in synovial fibroblasts from patients with rheumatoid arthritis. In those studies, RA-IgG leads to synovial fibroblast activation identical to that in TAO orbital fibroblasts. Those findings cannot be easily attributed to anti-TSHR antibodies such as TSIs. Another laboratory group presented strong evidence supporting the presence of activating anti–IGF-IR antibodies in a subset of patients with GD by using an assay involving HEK cells ^48^. Thus, in my view, considerably more investigation will be necessary before any definitive conclusions can be drawn concerning the nature of IGF-IR involvement in TAO.

## Clinical evidence for insulin-like growth factor-I receptor inhibition as therapy for thyroid-associated ophthalmopathy

Initial testing of the central hypothesis that IGF-IR is intimately involved in the pathogenesis of TAO was accomplished recently with the published report by Smith *et al*. ^44^. That study marks the completion of the first clinical trial of teprotumumab in patients with active, moderate to severe disease. The results of that study demonstrate unprecedented, profound reduction of *both* clinical activity score and proptosis, previously achieved only by surgical means. In total, 88 patients were enrolled in the placebo-controlled, double-masked, multinational trial in which the aggregate primary response comprised improvement of at least 2 points on a 7-point clinical activity score and reduction of proptosis of at least 2 mm at 24 weeks following initiation of the therapeutic phase in the study (more severely affected) eye. These improvements occurred in the absence of a similar magnitude of worsening of these parameters in the contralateral (fellow) eye. Secondary end-points included reduction of the clinical activity score of at least 2 points, reduction of proptosis of at least 2 mm (both measured as continuous variables), improvement in quality of life, and improvement in diplopia. The study design included a 24-week treatment period during which all participants received infusions of either placebo or active drug at 3-week intervals. The effects of the study drug were rapid, and many patients in the treatment group achieved primary end-point response within 6 weeks of their initial infusion. The safety profile was encouraging. The only side effect that could be unambiguously attributed to the drug was hyperglycemia, particularly in those patients with pre-existent diabetes mellitus. Glycemic control was easily restored with adjustment of the diabetes medications. Furthermore, carbohydrate intolerance in those individuals returned to baseline following the end of the treatment phase. Thus, it would appear that an inhibitory anti–IGF-IR antibody such as teprotumumab might play an important role in the therapy of active TAO. The findings of the trial suggest that the proposed mechanisms for anti–IGF-IR antibody activation of the TSHR/IGF-IR complex, coupled with the attenuation of signaling by teprotumumab, might at least in part underlie disease development. Interrupting this process might also interfere with disease progression and perhaps allow a “resetting of the immune clock”. This seems plausible in view of the reduction in proptosis observed in the recently concluded trial. Additional studies, conducted *in vivo*, *in vitro*, and potentially with experimental animal models, will be necessary to fully determine both disease mechanism and how teprotumumab is effecting its therapeutic benefit. Further inquiry will also be required to gain insight into the anatomic localization of drug action within the orbit. It remains unclear whether teprotumumab might be effective in stable disease. The remarkably rapid reduction in proptosis suggests that well-established disease might also be amenable to the drug’s beneficial effects. Based on the results of the trial, the US Food and Drug Administration designated teprotumumab with “breakthrough” status. The study outcome underscores the importance of conducting placebo-controlled trials in TAO and other relatively rare diseases. These are most likely to yield unambiguous results.

## Is B-cell depletion likely to reliably treat thyroid-associated ophthalmopathy?

Two recent small clinical studies investigated the effectiveness of anti-CD20 in active, moderate to severe TAO. The report by Stan *et al*. ^49^ described a comparison between rituximab and placebo which failed to demonstrate improvement in clinical activity score or proptosis attributable to the drug. The other, by Salvi *et al*. ^50^, found that rituximab was more effective than intravenous methylprednisolone in reducing clinical activity score but without clinically meaningful improvement in proptosis.

## Other treatment approaches with biological agents

With regard to the numerous biological agents already available and in wide use in other autoimmune diseases, interest has been generated to repurpose some for use in TAO. Initial experience with anti-tumor necrosis factor-alpha (anti-TNF-α) has generally proven discouraging, although the studies thus far conducted have been inadequately powered to draw firm conclusions. Tocilizumab, an interleukin-6 (IL-6) receptor antagonist, was examined in a single uncontrolled, prospective study involving 18 patients with TAO ^51^. The study suggests that the drug might prove effective in active disease, but its design makes any interpretation of the findings difficult. A well-controlled follow-up study might help clarify whether interruption of the IL-6 pathway is likely to yield clinical benefit. Certainly, ample evidence generated *in vitro* suggests that targeting this cytokine pathway might be a productive avenue to pursue.

Another potentially productive approach to therapy of active TAO involves interrupting the TSHR pathway. Two strategies have thus far been pursued. Several TSHR antagonizing monoclonal antibodies have been described ^52– 54^. Other laboratory investigators have developed small-molecule TSHR antagonists, including reverse agonists ^55– 57^. To my knowledge, none of these agents has reached the stage of clinical trials.

## Conclusions

Progress in understanding TAO over the past few years has yielded important detail concerning its relationship with GD occurring within the thyroid gland. This includes the apparent central importance of TSHR and TSI in development of both hyperthyroidism and ocular manifestations of the disease. Many questions remain unanswered. Among the most contentiously discussed is the involvement of IGF-IR in TAO. Widespread general agreement appears to exist that both physical and functional relationships exist between TSHR and IGF-IR ^42^ and some but not all aspects of these have been confirmed by other laboratory groups ^45^. Whether anti–IGF-IR antibodies develop in GD and how they might be involved in activating the receptor remain less well accepted. Emphatic declarations as to whether they exist and what their biological importance might be appear, in my view, to be wildly premature and potentially misleading. Some preliminary findings suggest that, besides IGF-IR, other therapeutic targets, including B-cell depletion, disrupting cytokine pathways, and interrupting TSHR signaling, will require careful consideration. Considerably more research will be required to fully assess the potential therapeutic landscape for TAO. Ultimately, strategies for promoting immune re-tolerization seem most promising since, in theory, they would spare patients the unpleasant prospects of long-term immunosuppression.

## Consent

The author thanks the patient whose image appears in Figure 1 for permission to publish. Written informed consent was obtained from the patient for the use of this image.
